# Prevalence and risk factors for Active Convulsive Epilepsy in Kintampo, Ghana

**DOI:** 10.11604/pamj.2015.21.29.6084

**Published:** 2015-05-13

**Authors:** Kenneth Ayuurebobi Ae-Ngibise, Bright Akpalu, Anthony Ngugi, Albert Akpalu, Francis Agbokey, Patrick Adjei, Damien Punguyire, Christian Bottomley, Charles Newton, Seth Owusu-Agyei

**Affiliations:** 1 Kintampo Health Research Centre, Ghana Health Service, Kintampo, Ghana; 2 Studies of the Epidemiology of Epilepsy in Demographic Surveillance Systems (SEEDS)-INDEPTH Network Accra, Ghana; 3 KEMRI/Wellcome Trust Research Programme, The Centre of Geographical Medicine Research- Coast, Kilifi, Kenya; 4 Population Health Sciences/Research Support Unit, Faculty of Health Sciences, Aga Khan University- East Africa, Nairobi, Kenya; 5 Department of Medicine, Korle Bu Teaching Hospital, Ghana; 6 Kintampo Municipal Hospital, Ghana Health Service, Ghana; 7 MRC Tropical Epidemiology Group, London School of Hygiene and Tropical Medicine, UK; 8 Department of Psychiatry, University of Oxford, United Kingdom

**Keywords:** Active Epilepsy, risk factors, sub-Saharan Africa, Ghana

## Abstract

**Introduction:**

Epilepsy is common in sub-Saharan Africa, but there is little data in West Africa, to develop public health measures for epilepsy in this region.

**Methods:**

We conducted a three-stage cross-sectional survey to determine the prevalence and risk factors for active convulsive epilepsy (ACE), and estimated the treatment gap in Kintampo situated in the middle of Ghana.

**Results:**

249 people with ACE were identified in a study population of 113,796 individuals. After adjusting for attrition and the sensitivity of the screening method, the prevalence of ACE was 10.1/1000 (95% Confidence Interval (95%CI) 9.5-10.7). In children aged <18 years, risk factors for ACE were: family history of seizures (OR=3.31; 95%CI: 1.83-5.96), abnormal delivery (OR=2.99; 95%CI: 1.07-8.34), problems after birth (OR=3.51; 95%CI: 1.02-12.06), and exposure to Onchocerca volvulus (OR=2.32; 95%CI: 1.12-4.78). In adults, a family history of seizures (OR=1.83; 95%CI: 1.05-3.20), never attended school (OR=11.68; 95%CI: 4.80-28.40), cassava consumption (OR=3.92; 95%CI: 1.14-13.54), pork consumption (OR=1.68; 95%CI: 1.09-2.58), history of snoring at least 3 nights per week (OR=3.40: 95%CI: 1.56-7.41), exposure to Toxoplasma gondii (OR=1.99; 95%CI: 1.15-3.45) and Onchocerca volvulus (OR=2.09: 95%CI: 1.29-3.40) were significant risk factors for the development of ACE. The self-reported treatment gap was 86.9% (95%CI: 83.5%-90.3%).

**Conclusion:**

ACE is common within the middle belt of Ghana and could be reduced with improved obstetric care and prevention of parasite infestations such as Onchocerca volvulus and Toxoplasma gondii.

## Introduction

Epilepsy is one of the most common neurological disorders worldwide, contributing one percent to the global burden of disease [1, 2]. About 69 million people worldwide are affected by this disorder with 90 percent of these individuals living in low- and middle-income countries [3]. In Africa, there are inadequate data on the burden, risk factors, and treatment gap on which to plan public health interventions. A review of the epidemiology and aetiology of epilepsy in sub-Saharan Africa (SSA) documented many descriptive studies of the aetiology of epilepsy, but only a few case-control studies [2, 4]. Most studies did not provide electroencephalography (EEG) or neuroimaging data. There are several population-based case-control studies in SSA [5–15] as well as more descriptive studies [16–20], that have highlighted the considerable heterogeneity in the prevalence of epilepsy and incidence of risk factors for epilepsy in the region. Parasitic infestations, such as *Onchocerca volvulus, Taenia solium and Toxoplasma gondii* are believed to increase the risk of epilepsy [21, 22], and these infections are common in most parts of SSA. Large community-based studies are needed to confirm these possible relationships, and to plan preventive/intervention measures. This study estimated the prevalence of, and identified risk factors for active convulsive epilepsy (ACE) in the two districts of Ghana with a Health and Demographic Surveillance Systems (HDSS). The study was conducted as part of a multi-site and multi-country study of ACE in SSA [6].

## Methods

**Study design**: a cross-sectional and case control studies was conducted to identify cases of ACE in the study area of the Kintampo Health Research Centre located in the middle belt of Ghana. This survey formed part of a large multi-centre study investigating the epidemiology of epilepsy in five sites in Africa with a HDSS, and was conducted between June 2010 and April 2011.

**Study area**: the study was carried out in the study area of Kintampo Health Research Centre (KHRC), which is located in the geographical middle of Ghana. The study area has a surface area of 7,162 square kilometers with the current resident population approximately 140,000 [23]. The main indigenous ethnic groups of the area are the Bonos and the Mos. There is, however, a large immigrant population mainly of Dagaabas, Dagombas and Konkombas from the three northern regions of Ghana as well as Ga Adangbes and Ewes with origins from southern Ghana. Twi is the commonest language spoken in the studied area. Community members engage in farming, predominantly of maize, yam and cassava. Livestock rearing of cattle, sheep, goats and poultry is also common, and some of the southerners fish on the Volta Lake. The vegetation is mainly of the forest-savannah transition type. There are two main seasons: the rainy season, which usually, start from March to November, and the dry season from December to February each year. The area is endemic for malaria [24] and onchocerciasis and some risk factors for toxocara and onchocerciasis are socio-economical [25]. The Kintampo Municipal Hospital and Jema Hospital are the two government hospitals in the Kintampo North Municipality and Kintampo South district respectively. They both have outpatient and inpatient facilities. The only psychiatric wing, manned by psychiatric nurses, is however, in the Kintampo North Municipal Hospital.

**The Survey Tools**: the study questionnaires on socio-demographic variables and historical risk factors for epilepsy [6] were translated into the widely-spoken local language (Twi) in the study area and finalised during training and pre-testing by project staff.

**Identification of cases of ACE**: a multistage approach was used to identify cases of ACE and has been published elsewhere [6]. In brief, KHRC maintains a Kintampo Demographic Surveillance System that has a database of all inhabitants of the study area. Firstly (Stage I), all members of the KHDSS population were screened for convulsion or fits using a two-item questionnaire administered by trained census fieldworkers [26]. The respondent was mainly the head or an adult member who have an in-depth knowledge about the household. During this stage, we sought to identify all people who had experienced a seizure in their lifetime. Secondly (Stage II), further detailed screening with a ten-question epilepsy questionnaire was administered by trained field supervisors who went to potential cases identified in the initial stage as ever having history of convulsion. At this stage, if the patient had at least two convulsions within their lifetime, with one in the last twelve months, he/she was referred to stage III for clinical evaluation and confirmation of ACE. Thirdly (Stage III), participants socio-demographic data was collected from caregivers. Clinical evaluation was performed by a trained clinician who made a diagnosis of ACE based upon a positive clinical history. The clinical examination focused at neurological deviations and blood was drawn from all patients for serological testing. The diagnosis of ACE was confirmed by a neurologist through the review of all information about the patient.

**Identification of controls**: age-matched controls (i.e. community members who did not have epilepsy) were randomly chosen from the Kintampo HDSS (KHDSS) database. Risk factors were determined using a questionnaire administered by a fieldworker to both cases of ACE and controls. Blood samples were taken from both cases and controls to determine exposure to parasitic infections (Onchocerca volvulus, Taenia solium, Plasmodium falciparum (schizont), Toxocara canis, Toxoplasma gondii) and HIV status. Exposure to infections was determined by detection of IgG antibodies to the parasitic antigens as well as HIV [27].

**Figure 1:**
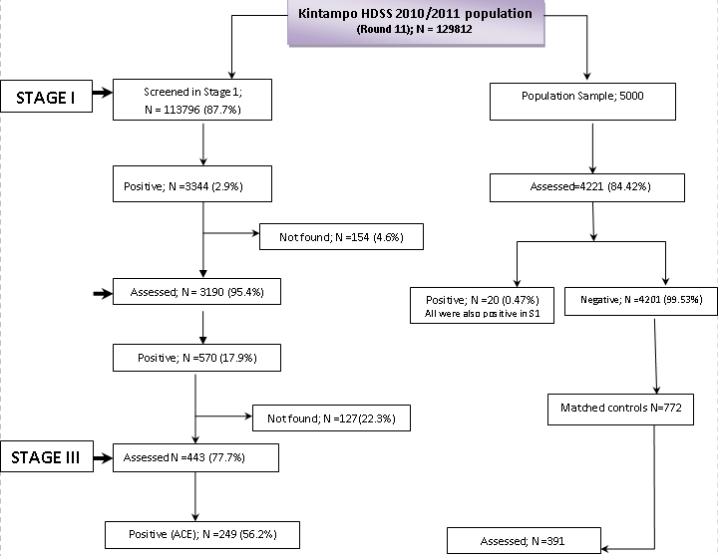
Study Flow chart showing the numbers at each stage of the study, 2011

**Population Sample**: a random sample of 5000 participants from the KHDSS were interviewed in stage II alongside the stage I positives from the census survey. This was done to compare the prevalence estimate from the two-stage method with that from the main three-stage study (Figure 1).

**Statistical Analysis**: data were double entered and verified in MySQL Version 5 open source database (Oracle Corporation, Redwood Shores, CA, USA) and all statistical analyses were carried out in STATA Version 11 (StataCorp, College Station, TX, USA). An unadjusted prevalence of ACE and 95% confidence interval (95%CI) was estimated as the number of cases of ACE confirmed in Stage III of the study divided by the total population screened in Stage I, expressed per 1,000 persons. Age, sex and sub-district specific prevalence of ACE were also estimated. Multiple imputation [28, 29] was used to reduce bias in the prevalence estimates due to attrition between stages in both the two- and three-stage surveys. Missing data were imputed at each stage of the survey using the outcome of the previous stage. Multiple imputation was carried out using the method of chained equations which was implemented using the “ice”command in STATA. The prevalence estimate was divided by 0.486 to account for the sensitivity (48.6%) of the three-stage screening methodology [30]. We report association between ACE, age, sex and sub-district. For each risk factor adjusted odd ratio (OR) were obtained by fitting logistic regression models that included age, sex, education (none, primary, or secondary and above), employment, marital status, and country as covariates as well as the risk factor of interest. Age was modelled as a fractional polynomial of degree two.

**Ethical Approvals**: written informed consent was obtained from all study participants. Unique reference codes were used to replace personal identifiers to ensure anonymity. Ethical approval was obtained from the Ghana Health Service Ethics Review Committee (Federal wide Assurance number -(FWA) 00020025), the Kintampo Health Research Centre Institutional Ethics Committee (FWA number 00011103) and the Ethics Committee of the Institute of Child Health, University College, London.

## Results

**Characteristics of the study population**: a total of 113,796 out of the 129,812 individuals in the KHDSS population (i.e. 87.7%) were screened in stage I. About 12.3% (16,016) of the targeted population in stage I were not met after three consecutive visits either migrated out of the study area or could not be found by the census fieldworkers. About 2.9% (3,344) of participants identified in stage I with history of convulsions of which 570 (17.9% of those positive in stage I) met the stage II referral criteria and were referred to the study clinic for detail clinical and neurological assessment (Figure 1). There were 20 individuals that were positive in stage II of the survey but who were missed by census fieldworkers in stage I. In stage III, 443 individuals were assessed, of whom 249 (56.2%) persons were confirmed as having ACE. The rest of the stage II referrals had febrile seizures. Demographic characteristics of the studied population are shown in Table 1.

**Table 1:** Age and sex distribution of study populations, 2011

Age-group	No	%
0-5	17,424	15.3
6-12	23,124	20.3
13-18	17,021	15.0
19-28	18,843	16.6
29-49	23,091	20.3
50+	14,286	12.6
**Sex**		
Female	57,888	50.9
Male	55,908	49.1
**Highest Education**		
None	62,356	54.8
Primary	19,750	17.4
Middle/continuation, JSS,	20,544	18.1
Technical, commercial, SSS	6,094	5.4
Post-middle college: Teacher, training, secretarial	223	0.2
Post-secondary: Nursing, teacher, polytechnic	4,498	3.95
University	331	0.29
**Total**	**113,796**	**100**

**The prevalence of Epilepsy in the KHDSS population**: the prevalence of convulsions in the entire KHDSS population was 29.2/1000 (95%CI: 28.3-30.2). The crude prevalence of ACE per 1000 people was 2.2/1000 (95%CI: 1.9-2.5), 4.9/1000 (95%CI: 4.4-5.3) when adjusted for attrition and 10.1/1000 (95%CI: 9.5-10.7) when adjusted for attrition and sensitivity. Males had higher prevalence of ACE (11.1/1000, 95%CI: 9.7-12.3) compared with females (9.1/1000, 95%CI: 7.8-10.3). The crude prevalence of ACE was highest in Kunsu, Jema and lowest in Kintampo sub districts (Table 2).

**Table 2:** Crude prevalence of ACE and parasitic infection by sub-district in the Kintampo area, 2011

Sub-District	Population	No of cases	Prevalence per 1000 people (95% CI)	Onchocerca volvulus	Toxoplasma gondii	Toxocara canis
				% control	% control	% control
Amoma	12,922	40	3.1 (2.2 - 4.2)	60.0(15/25)	48.0(12/25)	88.0(22/25)
Anyima	7,972	16	2.0 (1.1 - 3.3)	33.3(7/21)	57.1(12/21)	71.4(15/21)
Apesika	11,008	33	3.0 (2.1 - 4.2)	35.5(11/31)	71.0(22/31)	87.1(27/31)
Busuama	4,708	5	1.1 (0.3 - 2.5)	0.0(0/4)	100.0(4/4)	75.0(3/4)
Dawadawa	6,043	6	1.0 (0.4 - 2.2)	22.7(5/22)	68.2(15/22)	81.8(18/22)
Gulumpe	9,170	10	1.1 (0.5 - 2.0)	61.5(8/13)	61.5(8/13)	84.6(11/13)
Jema	19,204	60	3.1 (2.4 - 4.0)	34.6(28/81)	67.9(55/81)	81.5(66/81)
Kadelso	5,073	10	2.0 (0.9 - 3.6)	25.0(6/24)	58.3(14/24)	70.8(17/24)
Kintampo	38,797	22	0.6 (0.4 - 0.9)	30.2(19/63)	60.3(38/63)	73.2(46/63)
Kunsu	6,146	26	4.2 (2.8 - 6.2)	52.6(10/19)	63.2(12/19)	94.7(18/19)
Mansie	5,367	15	2.8 (1.6 - 4.6)	50.0(2/4)	75.0(3/4)	100.0(4/4)
New Longoro	3,402	6	1.8 (0.6 - 3.8)	0.0(0/1)	0.0(0/1)	100.0(1/1)
Total	129,812	249	1.9 (1.7 - 2.2)			

The denominators may not be the same as the number of cases because of missing data on Onchocerca volvulus, Toxoplasma gondii and Toxocara canis status. Also the controls were not matched to cases on sub-district hence the number of controls in some sub-districts is greater than the number of cases

**Age of onset of ACE**: the median age of onset of seizures was 8.0 (Interquartile Range, 2.8-15.0) years, and the median duration of seizures was 10.2 (Interquartile Range, 4.7-16.1) years.

**Risk Factor Analysis**: the analysis used data from 381 controls and 392 cases; 249 cases identified by the three-stage method and 143 cases identified from other sources including referrals during stage II screening, community members and health staff. In the logistic regression model for children below 18 years of age, family history of seizures (OR=3.31; 95%CI: 1.83-5.96), abnormal delivery (OR=2.99; 95%CI: 1.07-8.34), problems after birth (OR=3.51; 95%CI: 1.02-12.06), difficulties in feeding, crying or breathing after birth (OR=73.04; 95%CI: 9.81-544.07), and Onchocerca volvulus (OR=2.32; 95%CI: 1.12-4.78), were all significant risk factors for the onset of ACE (Table 3). Among adults, family history of seizures (OR=1.83; 95%CI: 1.05-3.20), history of no schooling (OR=11.68; 95%CI: 4.80-28.40), problems after birth (OR=8.74; 95%CI: 3.21-23.83), cassava consumption (OR=3.92; 95%CI: 1.14-13.54), pork consumption (OR=1.68; 95%CI: 1.09-2.58), history of snoring at least 3 nights / week) (OR=3.40: 95%CI: 1.56-7.41), Toxoplasma gondii IgG +ve (OR=1.99; 95%CI: 1.15-3.45) and onchocerca volvulus +ve (OR=2.09: 95%CI: 1.29-3.40) were all important risk factors for the development of ACE (Table 4)

**Table 3:** Regression analysis of risk factors for ACE in Children (<18 years)

	%	%	Unadjusted		Adjusted	
Risk Factor	Controls	Cases	OR (95% CI)	p-value	OR (95% CI)	p-value
Family history of seizure	14.0(24/172)	30.8(44/143)	2.74(1.52,5.02)	0.0003	3.31(1.83,5.96)	<0.001
Maternal seizures	0.6(1/172)	1.4(2/144)	2.41(0.12,142.89)	0.593	2.92(0.26,32.81)	0.386
Abnormal delivery	3.7(6/164)	9.2(13/141)	2.67(0.91,8.80)	0.057	2.99(1.07,8.34)	0.036
Abnormal antenatal period	11.5(19/165)	17.8(24/135)	1.66(0.83,3.38)	0.138	1.87(0.94,3.75)	0.075
Home delivery	71.3(119/167)	79.6(113/142)	1.57(0.90,2.77)	0.113	1.47(0.85,2.55)	0.173
Problems after birth	2.4(4/164)	7.7(11/142)	3.36(0.96,14.75)	0.036	3.51(1.02,12.06)	0.047
Difficulties feeding, crying or breathing	0.6(1/164)	31.6(43/136)	75.37(12.27,3062.68)	<0.001	73.04(9.81,544.07)	<0.001
Head injury	18.7(32/171)	25.0(36/144)	1.45(0.82,2.57)	0.216	1.45(0.82,2.54)	0.2
Malnourished	20.8(32/154)	12.5(15/120)	0.54(0.26,1.11)	0.077	0.56(0.28,1.12)	0.102
Eats cassava	100.0(170/170)	96.5(138/143)	0.00(0.00,0.63)	0.019	1.00(1.00,1.00)	
Dogs in household	48.3(83/172)	47.6(68/143)	0.97(0.61,1.55)	0.91	0.93(0.58,1.50)	0.777
Cats in household	33.1(57/172)	34.3(49/143)	1.05(0.64,1.73)	0.905	0.97(0.59,1.58)	0.896
Eats Pork	51.2(88/172)	53.8(77/143)	1.11(0.70,1.78)	0.652	1.12(0.70,1.78)	0.646
Malaria IgG +ve (schizont)	99.1(116/117)	100.0(93/93)	NA	1	NA	NA
Hospitalised with malaria or fever	0.0(0/173)	0.0(0/145)	NA	NA	NA	NA
Toxocara canis IgG4 +ve	13.7(16/117)	14.0(13/93)	1.03(0.43,2.42)	1	0.97(0.42,2.24)	0.935
Toxoplasma gondii IgG +ve	42.7(50/117)	51.6(48/93)	1.43(0.80,2.56)	0.213	1.06(0.58,1.94)	0.852
Taenia solium +ve	6.9(8/116)	4.3(4/93)	0.61(0.13,2.36)	0.554	0.40(0.10,1.66)	0.207
Onchocerca volvulus +ve	16.2(19/117)	33.3(31/93)	2.58(1.28,5.26)	0.005	2.32(1.12,4.78)	0.023
HIV +ve	20.5(24/117)	25.8(24/93)	1.35(0.67,2.71)	0.41	1.35(0.67,2.73)	0.395

Odds ration adjusted for: age, sex, maternal education, maternal marital status and employment of either parent. Fisher's exact test for unadjusted p-values.

**Table 4:** Regression analysis of risk factors for *ACE in Adult (≥18 years)*

	%	%	Unadjusted		Adjusted	
Risk Factor	Controls	Cases	OR (95% CI)	p-value	OR (95% CI)	p-value
Family history of seizure	13.0(27/207)	24.8(60/242)	2.20(1.30,3.77)	0.002	1.83(1.05,3.20)	0.034
Maternal seizures	0.0(0/207)	0.4(1/243)	NA	1	NA	NA
Abnormal delivery	8.1(16/198)	6.7(16/239)	0.82(0.37,1.80)	0.586	0.82(0.36,1.90)	0.646
Never Attended School	35.5 (72/203)	63.3(124/249)	2.4(1.67 – 3.39)	<0.001	11.68(4.80, 28.40)	<0.001
Home delivery	85.6(167/195)	86.0(208/242)	1.03(0.57,1.82)	1	1.07(0.58,1.99)	0.824
Problems after birth	3.1(6/196)	18.3(44/240)	7.11(2.92,20.82)	<0.0001	8.74(3.21,23.83)	<0.001
Head injury	4.3(9/208)	6.1(15/245)	1.44(0.58,3.82)	0.529	1.26(0.49,3.21)	0.633
Drinks alcohol	24.1(49/203)	27.3(66/242)	1.18(0.75,1.85)	0.514	1.51(0.91,2.52)	0.111
Eats cassava	93.2(193/207)	98.0(240/245)	3.48(1.16,12.54)	0.017	3.92(1.14,13.54)	0.031
Eats Pork	41.3(86/208)	49.0(119/243)	1.36(0.92,2.01)	0.108	1.68(1.09,2.58)	0.018
History of Snoring (3 nights /week)	25.2(41/303)	74.8(122/249)	5.0 (3.34 – 7.56)	<0.001	3.40(1.56, 7.41)	0.002
Uses drugs	1.9(4/206)	4.5(11/242)	2.40(0.70,10.50)	0.187	2.80(0.78,10.14)	0.116
Hypertension	2.5(5/202)	0.0(0/243)	0.00(0.00,0.63)	0.019	NA	NA
Stroke	0.0(0/202)	0.0(0/243)	NA	NA	NA	NA
Diabetes mellitus	0.5(1/201)	0.0(0/243)	0.00(0.00)	0.453	NA	NA
Malnourished	17.5(34/194)	18.6(41/221)	1.07(0.63,1.83)	0.8	0.97(0.53,1.77)	0.923
Dogs in household	49.5(103/208)	43.9(107/244)	0.80(0.54,1.17)	0.256	0.90(0.59,1.37)	0.616
Cats in household	30.3(63/208)	36.1(88/244)	1.30(0.86,1.97)	0.23	1.28(0.82,2.00)	0.275
Malaria IgG +ve (schizont)	100(175/175)	99.5(187/188)	0.00(0.00)	1	NA	NA
Hospitalised with malaria or fever	0.0(0/208)	0.0(0/245)	NA	NA	NA	NA
Toxocara canis IgG4 +ve	20.6(36/175)	24.5(46/188)	1.25(0.74,2.12)	0.383	1.26(0.72,2.21)	0.411
Toxoplasma gondii IgG + ve	67.4(118/175)	81.9(154/188)	2.19(1.31,3.68)	0.002	1.99(1.15,3.45)	0.014
Taenia solium +ve	4.0(7/174)	3.7(7/188)	0.92(0.27,3.15)	1	0.83(0.24,2.93)	0.774
Onchocerca volvulus +ve	32.0(56/175)	50.5(95/188)	2.17(1.39,3.41)	0.0004	2.09(1.29,3.40)	0.003
HIV +ve	30.9(54/175)	29.3(55/188)	0.93(0.58,1.49)	0.819	0.76(0.45,1.28)	0.303

Odds ratio adjusted for: age, sex, education, marital status and employment. Fisher's exact test for unadjusted p-values.

## Discussion

The study describes the prevalence as well as the risk factors for ACE in the Kintampo districts located in the middle belt of Ghana. ACE is noted to be widespread in the area with heterogeneity across the sub-districts. Antenatal/perinatal risk factors were associated with ACE in children and parasitic infections with adults.

**Prevalence**: the high prevalence of ACE reported in this study is similar to estimates from other countries in the sub-region [6, 9–11, 13] and within the range estimated for ACE in other low-income countries [6]. The prevalence was slightly higher among males compared with females. This finding has been reported in other studies [16] and might be due to greater risk taking behaviour of males compared with females even though head injury was not a significant risk factor in this study.

**Geographical Heterogeneity**: there crude prevalence of ACE varied across the sub-districts in the study area (Table 2). Apart from Kunsu sub-district, which is part of the Kintampo North municipality, the six sub-districts with the highest prevalence of ACE are situated in the Kintampo South district. There are many factors which may explain this difference. Firstly, there are fewer medical, particularly neurological services available in the South. Also, there are less Muslims in the Kintampo South compared with the Kintampo North, and since Muslims do not keep pigs or eat pork, this may contribute to explaining why there are fewer cases of ACE in this area. Jema and Kintampo that are nearer the main hospitals (Table 2) have a higher prevalence of ACE compared with other sub-districts. People with ACE in this area may be more likely to survive compared to other areas. This could be due to a more supervised delivery, early presentation and management of risk factors, e.g. treatment for parasitic infection among those closer to health facilities. Also routine deworming among school children is less likely to be effective among children in remote or sub-districts where school enrollment is usually low. In the adjoining district to Kintampo South district the prevalence of Onchocerca volvulus is high, where over 50% of the population lived in areas with a community microfilarial load in excess of 10 mf/snip [31] even though vector control programme have reduced transmission [32]. This may explain the relatively high prevalence of ACE in the Kintampo South District than in the Kintampo North Municipality.

**Risk factors for ACE**: there was a significant association between a history of family seizures and ACE. This association has been reported by Prischich et al. [16]. Nevertheless, the effect of family history might be overestimated because people with active epilepsy may be more aware of other family members who are affected by seizures compared with those who do not have epilepsy. Also, people with ACE may have similar exposure to parasitic infections compared with those who do not have epilepsy. Antenatal difficulties (Table 3) were associated with the development of active epilepsy. Studies have reported that birth asphyxia is a preventable risk factor for epilepsy [6, 33]. The percentage of home deliveries in Ghana is now 42% [34], and home deliveries in Kintampo is about 30% [35]. Onchocerciasis infection might be a risk factor for the development of ACE in adults within the study area since other studies have found an association between onchocerciasis and epilepsy [22, 36, 37]. However, it is also possible that people with epilepsy have increased exposure to parasites compared with those who do not just because of their vulnerability. There have been a number of programmes in West Africa, including Ghana, for the control and eradication of onchocerciasis [38]. In Ghana, vector control has virtually ceased, except limited activities associated with the construction of the Bui Dam, a hydroelectric dam located within 71.4 KM of the study area [39, 40]. Resistant adult parasite populations, which were not responding as expected to ivermectin, have emerged [41, 42]. A high rate of repopulation of skin with microfilariae will allow parasite transmission, possibly with ivermectin-resistant O. volvulus, which could eventually lead to recrudescence of the disease [41]. Further research is needed to improve monitoring and establish thresholds that determine when control is needed. In this study, history of head injury, exposure to infections such as malaria (as measured by schizont antibodies), taenia and HIV were not significant risk factors for the development of active convulsive epilepsy. Kintampo is a malaria endemic area [43] so that nearly everybody is exposed to malaria. Other parasitic infections such as toxocara and toxoplasmosis have been documented elsewhere to be associated with epilepsy [44–46], even though their relationship with epilepsy in SSA has not been properly documented [44]. The prevalence of taenia and head injury is relatively low in this study even though other studies in West Africa, such as Nigeria have reported an association between head injury and epilepsy [8, 45, 46]. This study found consumption of cassava to be significantly associated with active epilepsy in adults. Our study area is predominantly rural and many inhabitants cultivate rooted carbohydrate crops such as cassava and yam. Cassava consumption might be a marker of poverty which is strongly associated with ACE, but cassava consumption maybe related to ACE through malnutrition. This risk might be due to the consumption of freshly uprooted cassava which has a high concentration of cyanide compared to a day or two after uprooting [47]. Surprisingly, snoring at least three times per night in a week was a risk factor for developing active epilepsy. Some studies have reported the presence of snoring among epileptic patients [48, 49]. Previous studies have reported association of obstructive sleep apnea and epilepsy [50].

**Limitations of the study**: as the respondents were asked to recall exposure to risk factors in the past, this could have resulted in recall bias of some of the risk factors such as abnormal delivery, difficulties feeding, crying or breathing after birth. Having been involved in previous clinical trials, the community has been sensitised to recall of health-related issues and may not be far from being correct in the recalls made in this study.

## Conclusion

Epilepsy is a common neurological disorder in this part of Ghana. Active convulsive epilepsy is associated with family history of epilepsy, history of snoring, perinatal difficulties and parasitic infection. The major preventable risk factors for children are perinatal and post natal difficulties and exposure to Onchocerca volvulus. Adult preventable risk factors included no formal education, problems after birth, cassava and pork consumption, and parasitic infections such as T. gondii and O. volvulus. Adequate and proper prenatal care aimed at avoiding problems during pregnancy and perinatal measures could reduce complications that may result in epilepsy. Also, control measures for onchocerciasis and the strengthening of the health systems to include information about the risk factors for ACE in their outreach programmes could reduce the prevalence of epilepsy in Ghana.

**Future Research**: there is the need to conduct further studies on the incidence of ACE, excess mortality in people diagnosed as having ACE, and the risk factors for death among people with ACE. Also, the relationship between snoring and epilepsy, cassava consumption and epilepsy need to be investigated.
